# Characteristics of Patients Experiencing a Flare of Generalized Pustular Psoriasis: A Multicenter Observational Study

**DOI:** 10.3390/vaccines11040740

**Published:** 2023-03-27

**Authors:** Francesco Bellinato, Paolo Gisondi, Angelo Valerio Marzano, Stefano Piaserico, Clara De Simone, Giovanni Damiani, Giuseppe Argenziano, Marina Venturini, Paolo Dapavo, Antonio Costanzo, Matteo Megna, Francesca Prignano, Martina Burlando, Francesca Satolli, Andrea Carugno, Elena Pezzolo, Marco Romanelli, Aldo Cuccia, Giampiero Girolomoni

**Affiliations:** 1 Section of Dermatology and Venereology, Department of Medicine, University of Verona, Piazzale A. Stefani 1, 37126 Verona, Italy; 2 Dermatology Unit, Fondazione IRCCS Ca’ Granda Ospedale Maggiore Policlinico, 20122 Milan, Italy; 3 Department of Pathophysiology and Transplantation, Università degli Studi di Milano, 20133 Milan, Italy; 4 Dermatology Unit, Department of Medicine, University of Padua, 35122 Padua, Italy; 5 Dermatologia, Dipartimento Scienze Mediche e Chirurgiche, Fondazione Policlinico Universitario A. Gemelli IRCCS, 00168 Rome, Italy; 6 Dermatologia, Dipartimento Universitario Di Medicina e Chirurgia Traslazionale, Università Cattolica Del Sacro Cuore, 20123 Rome, Italy; 7 Department of Biomedical, Surgical and Dental Sciences, University of Milan, 20126 Milan, Italy; 8 Unit of Dermatology and Cosmetology, IRCCS San Raffaele Hospital, 20132 Milan, Italy; 9 Dermatology Unit, Università Degli Studi Della Campania L. Vanvitelli, 80131 Naples, Italy; 10 Department of Dermatology, University of Brescia at ASST-Spedali Civili, 25121 Brescia, Italy; 11 Section of Dermatology, Department of Medical Sciences, University of Turin, 10124 Turin, Italy; 12 Dermatology Unit, Department of Biomedical Sciences, Humanitas University, 20072 Pieve Emanuele, Italy; 13 Humanitas Clinical and Research Center, Scientific Institute for Research, Hospitalization and Healthcare, 20089 Rozzano, Italy; 14 Section of Dermatology, Department of Medicine and Surgery, University of Naples Federico II, 80138 Naples, Italy; 15 Section of Dermatology, Department of Health Sciences, University of Florence, 50125 Florence, Italy; 16 Department of Dermatology, Dipartimento Della Salute-DiSSal, University of Genoa, 16126 Genoa, Italy; 17 IRCCS, Ospedale Policlinico San Martino, 16132 Genoa, Italy; 18 Section of Dermatology, Department of Medicine and Surgery, University of Parma, 43100 Parma, Italy; 19 Dermatology Unit, ASST Papa Giovanni XXIII Hospital, 24127 Bergamo, Italy; 20 Ph.D. Program in Molecular and Translational Medicine (DIMET), University of Milan-Bicocca, 20126 Milan, Italy; 21 Dermatology Unit, San Bortolo Hospital, 36100 Vicenza, Italy; 22 Department of Dermatology, University of Pisa, 56124 Pisa, Italy; 23 Unit of Dermatology, San Donato Hospital, 52100 Arezzo, Italy

**Keywords:** generalized pustular psoriasis, flare, psoriasis

## Abstract

Background: Generalized pustular psoriasis (GPP) is a rare, severe inflammatory skin disease characterized by recurrent episodes of flares. Characteristics of patients experiencing a flare are hardly described in a real-life setting. The aim of the study is to investigate the clinical characteristics of patients experiencing a flare of GPP. Methods: Multicenter retrospective observational study on consecutive patients experiencing a flare of GPP between 2018 and 2022. Disease severity and quality of life were assessed by Generalized Pustular Psoriasis Area, Body Surface Area (BSA), and Severity Index (GPPASI), and Dermatology life quality index (DLQI) questionnaire, respectively. Visual analogue scale (VAS) of itch and pain, triggers, complications, comorbidities, pharmacological therapies, and outcome were collected. Results: A total of 66 patients, 45 (68.2%) females, mean age 58.1 ± 14.9 years, were included. The GPPASI, BSA, and DLQI were 22.9 ± 13.5 (mean ± standard deviation), 47.9 ± 29.1, and 21.0 ± 5.0, respectively. The VAS of itch and pain were 6.2 ± 3.3 and 6.2 ± 3.0, respectively. Fever (>38 °C) and leukocytosis (WBC > 12 × 10^9^/L) were found in 26 (39.4%) and 39 (59.1%) patients, respectively. Precipitating triggers were identified in 24 (36.3%) and included infections (15.9%), drugs (10.6%), stressful life events (7.6%), and corticosteroids withdrawal (3.0%). Fourteen (21.2%) patients were hospitalized because of complications including infections in 9 (13.6%) leading to death in one case and hepatitis in 3 (4.5%). Conclusions: GPP flares can be severe and cause severe pain and itch with significant impact on the quality of life. In about one-third of patients the flare may have a persistent course and, with complications, lead to hospitalization.

## 1. Introduction

Generalized pustular psoriasis (GPP) is defined as primary, sterile, macroscopically visible pustules on non-acral skin (excluding cases where pustulation is restricted to psoriatic plaques) [1,2,3,4]. GPP can occur with or without systemic inflammation, with or without plaque psoriasis, and can be either a relapsing (>1 episode) or persistent (>3 months) condition [5]. There is a large degree of variability in the prevalence of GPP in the published literature, particularly between Western and Asian countries, reflecting different methodologies, populations, and definitions of GPP, and the inclusion, or lack of differentiation from localized variants of pustular psoriasis. The point prevalence, annual prevalence and annual incidence of GPP was estimated in Sweden at 9.1 per 100,000, 1.5 per 100,000, and 1.53 per 100,000, respectively [6]. A national French questionnaire-based study estimated an annual prevalence of GPP at 0.18 cases per 100,000 [7]. A review of a national insurance claims database in the Republic of Korea reported a prevalence of 12 cases per 100,000 persons [8]. Another Japanese survey of 575 community center hospitals found a prevalence of 0.74 patients per 100,000 persons [9].

GPP has been observed in 9% of incident pustular psoriasis patients, being much rarer than palmoplantar pustulosis (PPP) that occurs in 83% of cases [10]. GPP results from dysregulation of the innate immune system, particularly involving the interleukin (IL)-36 inflammatory pathway, induction of inflammatory keratinocyte responses, and recruitment of neutrophils [11]. Mutations in IL36RN, CARD13, AP1S3, MPO, TNIP1, SERPINA3, and SERPINA1 have been shown to be associated with GPP, among which loss-of-function mutation in IL36RN is the dominant mutation with the highest prevalence. IL-36RN, MPO, and SERPINA 1/3 mutations lead to the upregulation of IL-36 signalling, which further activates the downstream proinflammatory NF-κB and MAPK pathways. AP1S3 and TNIP1 loss-of-function mutations and CARD14 gain-of-function mutations are involved in IL-36 signalling by hyperactivating the NF-κB pathway. The upregulated IL-36 signalling promotes the proliferation of IL-17-producing CD4+Th17 cells, and these cytokines secreted by infiltrating CD4+T cells further propagate these inflammatory cycles by inducing expression of IL-36 and other inflammatory mediators. The interaction of the IL-36 pathway and the IL-23/IL-17 axis underlies the immunological disturbances of GPP, indicating that innate and adaptive immune responses intertwine in the pathogenesis of GPP [12].

GPP flares are acute events, which can be very impactful and occasionally associated with mortality due to complications, including sepsis, acute respiratory distress syndrome, renal failure, and congestive heart failure [13]. Various triggers, such as corticosteroids withdrawal, drugs, stressful life events, and infections, have been reported [13]. Given the rarity of the disease in Europe compared to Japan and Asian countries [6,7,8,9,14,15], the clinical characteristics of patients experiencing a flare of GPP are hardly reported. The aim of this study was to investigate the clinical characteristics of a series of patients experiencing a flare of GPP in a real-life multicenter setting.

## 2. Material and Methods

This retrospective observational study from 16 dermatology centres in Italy included a consecutive series of 66 patients experiencing a flare of GPP between 1 January 2018 and 31 December 2022. Data were collected on a shared case report form that has been completed by a selected group of dermatologists who have recognized experience in the diagnosis and treatment of GPP. ERASPEN network criteria were applied to classify GPP (von Zumbusch type) [2]. In particular, GPP was defined in case of macroscopically visible sterile pustules occurring on non-acral skin and not within psoriasis plaques, with at least one relapse or persisting for over 3 months. The diagnosis of GPP was clinically confirmed by the dermatologist and was supported by histological examination when deemed necessary. Flares of GPP were defined as acute appearance of pustules affecting large body areas on an erythematous skin that could be accompanied by systemic symptoms, such as fever, leukocytosis, and fatigue [2]. Inclusion criteria was the occurrence of the GPP flare during the observational period (i.e., between 2018 and 2022) and given consensus to the anonymous collection of data. Exclusion criteria were localized forms of pustular psoriasis (i.e., acrodermatitis continua of Hallopeau [ACH] and palmoplantar pustular psoriasis [PPP]). PPP was defined only on a clinical basis, i.e., in the presence of chronic pustular dermatitis involving the palms and soles characterized by vesicles, pustules, erythema, lichenification, and abnormal desquamation according to ERASPEN criteria [2].

Demographic and clinical characteristics of the patients including age, gender, body mass index (BMI), personal and family history of chronic plaque psoriasis, smoking habit, age of GPP onset, history of PPP and ACH, disease course of GPP (relapsing, i.e., had had more than a single episode and/or persistent; i.e., flare persisting for over 3 months), and the number of previous flares were collected. Disease severity at flare was assessed by Generalized Pustular Psoriasis Area and Severity Index (GPPASI) ranging from 0 to 72, and Generalized Pustular Psoriasis Physician Global Assessment (GPPGA) ranging from 0 to 5 [16]. The presence of fever (≥38 °C) and leukocytosis (white blood cells > 12 × 10^9^/L) were also investigated [2]. Disease localization was estimated by proportion of the body surface area involvement (BSA %) and detailed for body regions, including mucosal and nail involvement. Oral and genital mucosal involvement was confirmed in the case of geographic tongue, and erythema/pustules in the genital area, respectively. Nail involvement was recognized in case of any of the following manifestations: leukonychia, onycholysis, salmon patch, splinter haemorrhage, hyperkeratotic lamina, subungual hyperkeratosis, onycholysis, and/or pitting. Subjective symptoms of itch and pain were assessed by Visual Analogue Scale (range 0–10). Dermatology life quality index (DLQI) questionnaire was also administered [17]. Concomitant psoriatic arthritis was assessed according to the CASPAR criteria [18]. History of previous medical diagnosis of and/or treatment of type 2 diabetes, obesity, arterial hypertension, dyslipidaemia, hyperuricemia, and malignancy were investigated. The Charlson Comorbidity Index (CCI), which identifies different comorbidities that are associated with mortality, was calculated [16]. CCI final score ranges from 0 (no disease burden) to 24 (maximal disease burden), and the severity of comorbidity can be categorized into three grades: mild, with CCI scores of 1–2; moderate, with CCI scores of 3–4; and severe, with CCI scores ≥5. The risk for mortality increases with CCI scores [19,20]. Finally, possible triggers, complications, and number and type of treatments prescribed for managing the flare were collected. The clinical characteristics of the patients, the number of pharmacological systemic treatments that patients were prescribed because of the GPP flare; their outcomes reflected when the patient had the flare of GPP.

The present study was conducted in accordance with the Declaration of Helsinki, initially published in 1964, on Ethical Principles for Medical Research Involving Human Subjects and approval by the local ethical committees.

### Statistical Analysis

Percentages were given for categorical variables, mean, and standard deviation, or median and interquartile range were given for normally or non-normally distributed continuous variables, respectively. Subgroup analyses were performed with the Mann–Whitney U Test to compare continuous variables and Fisher’s exact test was used for categorical variables. The associations between continuous variables of interest were tested by linear regression. The statistical alpha significance level was accepted as *p* < 0.05. Statistical analysis was performed using STATA (version 13 StataCorp, College Station, TX, USA).

## 3. Results

Descriptive characteristics of the study population are summarized in Table 1. A total of 66 patients were collected, 45 (68.2%) were female (M:F ratio 0.47, 21/45) with an age at flare of 58.1 ± 14.9 years, ranging from 14 to 91; the mean BMI was 26.3 ± 4.4 kg/m^2^; and 15 (22.7%) were current smokers. A total of 39 (59.1%) had personal history of chronic plaque psoriasis and 17 (25.8%) had a positive family history of psoriasis. Disease age of onset did not differ between patients with history of plaque psoriasis vs. those without. The CCI was 2.2 ± 1.9, being arterial hypertension, dyslipidaemia, and psoriatic arthritis, the more frequent comorbidities affecting 28 (42.4%), 18 (27.3%), and 12 (18.2%) of patients, respectively. In particular, peripheral arthritis and dactylitis were found in 9 (13.6%) and 3 (4.5%) patients, respectively. Regarding disease course, most of the patients (52 out of 66, 78.8%) had history of relapsing GPP course, and in 20 (30.3%) the flare was persistent, lasting more than 3 months. Considering the frequency of flare, 6 out of 66 (9.1%) had more than one single episode per year, and 4 (6.1%) more than two. Of note, comparing disease severity of the patients with frequent flares (i.e., more than 1 flare every 5 years) versus those with rare flares (i.e., less than 1 flare every 5 years), we found that the former had higher GPPASI compared to latter, 37 (23–43) vs. 19 (10–30); Mann–Whitney U Test *p* = 0.007.

Personal history of PPP and ACH were reported in 24 (36.4%) and in 8 (12.1%) patients, respectively. No differences were found in the prevalence of PPP and ACH in patients with a history of plaque psoriasis versus those without. Severity, localization, triggers, and complications of the GPP flare are summarized in Table 2. In particular, the GPPASI was 22.9 ± 13.5 (mean ± SD), GPPGA was 3.3 ± 0.8, and BSA 47.9 ± 29.1%. The VAS of itch and pain was 6.2 ± 3.3 and 6.2 ± 3.0, respectively. The DLQI was 21.0 ± 4.9. Of note, at linear regression analysis DLQI correlated with GPPASI score (β = 0.25, *p* < 0.001) and VAS pain (β = 0.95, *p* < 0.001). A total of 26 (39.4%) patients had fever and 39 (59.1%) leukocytosis. The most common disease localizations were the trunk (95.5%), superior (92.4%) and inferior limbs (84.8%), folds (60.6%), and head-neck area (57.6%). Nail involvement was found in 33.3% of patients, The most common manifestation was leukonychia and onycholysis affecting 40% and 32% of patients, respectively. Geographic tongue was found in 7 (10.6%), and genital area involvement in 32 (48.5%) of patients. A possible trigger was identified in 25 (37.9%) of the flares, including upper respiratory tract infections (15.2%), drugs, generally antibiotics (10.6%), stressful life events (7.6%), corticosteroids withdrawal (3.0%), hypocalcaemia (1.5%), and pregnancy (1.5%). The flare needed hospitalization when complicated by infection (13.6%), hepatitis (4.5%). In particular, a case of COVID-19 pneumonia was fatal in one patient aged 84 with ischemic cardiopathy. No differences were found in severity, localization triggers, complications, and flare patterns between patients with history of plaque psoriasis versus those without.

A mean of 2.4 ± 1.1 different systemic treatments (range 1–5) were prescribed to manage the flares. In this series, the most common prescribed treatments were systemic corticosteroids alone in 46 (69.7%) or in combination with systemic retinoids in 24 (36.4%) cases. The last common treatment lines were: ixekizumab (22.7%), risankizumab (16.6%), secukinumab (19.7%), adalimumab (9.1%), guselkumab (3%), tildrakizumab (3%), and brodalumab (3%), followed by few cases treated with non-biologic treatments (cyclosporine and methotrexate), NB-UVB and PUVA phototherapy. Such treatments were generally in combination with systemic corticosteroids and retinoids and associated with short hospitalization stay and complete resolution of the pustules. In 44 out of 66 (66.7%) included patients, the average treatment time course was 4.5 ± 1.2 weeks (mean ± SD).

## 4. Discussion

In this multicentre study, we describe the clinical characteristics and severity of a series of consecutive patients experiencing a flare of GPP, defined according to the ERASPEN criteria. The major findings of the study include that the flare affects more frequently females with a M:F ratio of 0.47 (21/45), aged between 50 and 60 years, which is consistent with different Asian, European, and American series [6,7,8,9,21,22,23,24,25,26,27,28,29,30,31,32]. For example, in recent Turkish study of 156 GPP patients by Polat AK et al. a M:F ratio of 0.6 was estimated [13]. Although GPP can occur in children, it typically emerges in adulthood and according to the existing literature the onset age can range from 20 years to 58 years. In our sample, we found an average of 58.1 ± 14.9 years, which is consistent with previous studies [21,22,23,24,25,26,27,28,29,30,31,32]. The history of plaque psoriasis in GPP patients broadly varies between 30–70.4%, usually preceding GPP of some years [21,22,23,24,25,26,27,28,29,30,31,32]. In the present study, 39 out of 66 (59%) patients had a personal history of chronic plaque psoriasis. No significant differences were found in clinical features of disease (i.e., onset age and severity) between patients with personal history of plaque psoriasis vs. those without. Accumulating genetic, transcriptomic, and clinical evidence supports the idea that GPP and plaque psoriasis are distinct diseases, but they can coexist [33].

Both European and Japanese diagnostic criteria highlight the relapsing nature of GPP, which can be triggered by several events [1]. Triggers act mainly by dysregulating the IL-36 axis in epidermal keratinocytes through IL-17C [34]. In this study, the precipitating factor was identified in 37.8% (25/66) of the cases, including infections in 15.2% (10/66), drugs in 10.6% (7/66), stressful events in 7.6% (5/66), corticosteroids withdrawal in 3.0% (2/66), hypocalcaemia in 1.5% (1/66), and pregnancy in 1.5% (1/66). Among drugs, antibiotics, terbinafine, TNF-alpha inhibitors, and corticosteroids withdrawal have been reported [13,35]. Different infections, including upper respiratory infections and COVID-19 have been associated with GPP exacerbation. The role of stressful life events in triggering GPP is still controversial [36].

The novelty of our study is the assessment of GPP disease severity at flare through GPPASI, which was on average 22.8 ± 13.5, with an involvement of almost half BSA, including nail and genitals involvement, with moderate-to-severe itch and skin pain (VAS > 6). Our result confirms the significant impact of GPP on the quality of life, as we found a mean DLQI of 21.0 ± 4.9. There have been few studies evaluating the quality of life in GPP patients. Polat AK et al. reported DLQI lower scores (11.4 ± 9.8) [13]. Extensive cutaneous involvement, itch and pain, and systemic symptoms and comorbidities, contribute to the negative impact of quality of life. The flare presentation in our series was characterized by high fever of 39.4% and leukocytosis in 59.1% (data not shown in tables). In the literature, high fever has been reported in 24–96% of patients during GPP flare and leukocytosis (with neutrophilia) in 30–70%. Systemic inflammation can be also associated with fatigue, malaise, diarrhoea, laboratory abnormalities (raised C-reactive protein and erythrocyte sedimentation rate), and extracutaneous neutrophilic inflammatory involvement, including cholestasis, arthritis, interstitial pneumonia, oral lesions, and acute renal failure [1,2,3,4].

Interestingly enough, patients with severe disease showed more frequent flares. In detail, almost 80% of the patients had recurrent flares, but only less than 10% had more than one single episode per year. Few studies assessed the GPP course in terms of frequency of flares. A study using the French National Health Data System database showed that most patients with GPP had no flare or only one episode per year [7]. In particular, 79.4% had only 1 flare and 17.6% had 2 to 4 flares. Flare duration ranged broadly from few weeks to persistent forms in 30% of the cases, probably reflecting disease severity, timing, treatment efficacy, and comorbidities. In a large retrospective cohort study based on electronic records in US, patients with GPP flares had 34% higher mean CCI score (2.80 ± 3.11 vs. 2.09 ± 2.52) compared to those without. In addition, they had higher inpatients and emergency department visits and higher use of all treatment [37]. In our study, we found a mean CCI score of 2.2 ± 1.9, reflecting a similar comorbidity burden.

The rarity of GPP makes clinical trials challenging and treatment recommendations are based on insufficient evidence [38]. These include corticosteroids, cyclosporin, methotrexate, retinoids, and infliximab [2,36,38]. A mean of 2.4 ± 1.1 different systemic treatments (range 1–5) were prescribed to manage the flare in our case series, generally starting with systemic corticosteroids and/or retinoids. To our knowledge, data about the proportion of multi-failure GPP are scarce. In a Turkish cross-sectional study, 36% of the patients with GPP flare were lesion-free under treatment [13]. Phase 3 studies in Japanese patients showed the efficacy of target biological therapies targeting IL-17 and IL-23 (namely, ixeziumab, secukinumab, brodalumab, guselkumab, and risankizumab) in GPP [39,40,41,42,43,44]. Nonetheless, it was estimated that up to 10% may not respond to treatment [45]. Emerging therapies targeting IL-36R, such as spesolimab and imsidolimab, showed promising results in clinical trials in terms of efficacy and safety and may improve the management of GPP in the future [46]. In fact, approximately 20% of the flares needed hospitalization due to complications, with a mortality rate of 1.6%. It was estimated that a patient with GPP will require hospitalization for a flare at least once every 5 years, with a duration of stay of 10 to 14 days [21]. According to published data, the mortality rate ranges between 0% to 16%. Our low rate could be associated with the high efficacy of new target therapies, as confirmed also by Polat AK et al. that found no mortality rate in a large group of 156 patients with GPP flare [13].

The study has some limitations and strengths. The retrospective design resulted in recall bias, the longitudinal follow-up is relatively short to address properly the disease course and causative inferences between treatments and disease course cannot be drawn and the sample size relatively small. Finally, we did not test for the presence of genetic mutations (IL36RN, CARD14, and AP1S3). In fact, a recent Malaysian study found that IL36RN disease alleles are associated with a severe and early onset clinical phenotype [47]. Choon SE et al. also found that patients with true PPP hardly ever had IL36RN mutations, while ACH is common in patients with IL36RN [47]. Nonetheless, this was a multicentre study collecting GPP cases according to the ERASPEN criteria definition and emphasizing their flare phase. Future longitudinal studies are needed to better understand GPP disease course and its burden of comorbidities. In conclusion, GPP flares can be severe and cause severe pain and itch with significant impact on the quality of life. A proportion of patients may have persistent disease and complications requiring hospitalization. Given the rarity of GPP and the unpredictable course of the disease, a better understanding of GPP flare is crucial to achieve the optimal management strategy.

## Figures and Tables

**Table 1:** Characteristics of the patients experiencing a flare of generalised pustular psoriasis.

Number of Patients	N = 66
Age, mean ± SD years	58.1 ± 14.9
Gender, female N (%)	45 (68.2)
Age of GPP onset, mean ± SD years	49.5 ± 14.5
BMI, mean ± SD kg/m^2^	26.3 ± 4.4
History of chronic plaque psoriasis	39 (59.1)
Family history of plaque psoriasis	17 (25.8)
History of palmoplantar pustular psoriasis	24 (36.4)
History of acrodermatitis continua of Hallopeau	8 (12.1)
**Smoking Habit**	
Current	15 (22.7)
Past	12 (18.2)
Never	39 (59.1)
**History of GPP Course**	
Relapsing (>1 episode lifetime)	52 (78.8)
≥1 flare per year	6 (9.1)
≥2 flares per year	4 (6.1)
Persistent (>3 months)	20 (30.3)
**Comorbidities**	
Charlson comorbidity index	2.2 ± 1.9
Arterial hypertension	28 (42.4)
Hyperlipidaemia	18 (27.3)
Psoriatic arthritis	12 (18.2)
Peripheral joint	9 (13.6)
Dactylitis	3 (4.5)
Type 2 diabetes mellitus	10 (15.2)
Ischaemic heart disease	8 (12.1)
Hyperuricemia	4 (6.1)
Obesity	6 (9.1)
History of malignancy	5 (7.6)

**Table 2:** Severity, localization, triggers, and complications of GPP flare.

Disease Severity	
GPPASI, mean ± SD	22.9 ± 13.5
GPPGA, mean ± SD	3.3 ± 0.8
BSA, mean ± SD	47.9 ± 29.1
VAS itch, mean ± SD	6.2 ± 3.3
VAS pain, mean ± SD	6.2 ± 3.0
DLQI, mean ± SD	21.0 ± 4.9
Fever (>38 °C)	26 (39.4)
Leucocytosis (WBC > 12 × 10^9^/L)	39 (59.1)
**Disease Localization**	
Trunk	63 (95.5)
Superior limbs	61 (92.4)
Inferior limbs	56 (84.8)
Folds	40 (60.6)
Head-neck	38 (57.6)
Genital area	32 (48.5)
Nails	22 (33.3)
Oral mucosa	7 (10.6)
**Triggers**	
Infections	10 (15.2)
Drugs	7 (10.6)
Stressful events	5 (7.6)
Corticosteroids withdrawal	2 (3.0)
Hypocalcaemia	1 (1.5)
Pregnancy	1 (1.5)
**Complications**	
Infections	9 (13.6)
Hepatitis	3 (4.5)
Death	1 (1.5)

## Data Availability

No additional data available.
